# Personalising treatment plan quality review with knowledge-based planning in the TROG 15.03 trial for stereotactic ablative body radiotherapy in primary kidney cancer

**DOI:** 10.1186/s13014-021-01820-7

**Published:** 2021-08-03

**Authors:** Nicholas Hardcastle, Olivia Cook, Xenia Ray, Alisha Moore, Kevin L. Moore, David Pryor, Alana Rossi, Farshad Foroudi, Tomas Kron, Shankar Siva

**Affiliations:** 1 Physical Sciences, Peter MacCallum Cancer Centre, 305 Grattan St, Melbourne, VIC 3000 Australia; 2 Centre for Medical Radiation Physics, University of Wollongong, Wollongong, Australia; 3 Trans Tasman Radiation Oncology Group, Newcastle, Australia; 4 Department of Radiation Medicine and Applied Sciences, University of California San Diego, San Diego, USA; 5 Department of Radiation Oncology, Princess Alexandra Hospital, Brisbane, Australia; 6 Olivia Newton, John Cancer Centre at Austin Health, Heidelberg, Australia; 7 Department of Oncology, Sir Peter MacCallum, University of Melbourne, Parkville, Australia; 8 Radiation Oncology, Peter MacCallum Cancer Centre, Melbourne, Australia

**Keywords:** SABR, SBRT, Kidney, Knowledge based planning, Clinical trial, Quality assurance, Renal cell carcinoma

## Abstract

**Introduction:**

Quality assurance (QA) of treatment plans in clinical trials improves protocol compliance and patient outcomes. Retrospective use of knowledge-based-planning (KBP) in clinical trials has demonstrated improved treatment plan quality and consistency. We report the results of prospective use of KBP for real-time QA of treatment plan quality in the TROG 15.03 FASTRACK II trial, which evaluates efficacy of stereotactic ablative body radiotherapy (SABR) for kidney cancer.

**Methods:**

A KBP model was generated based on single institution data. For each patient in the KBP phase (open to the last 31 patients in the trial), the treating centre submitted treatment plans 7 days prior to treatment. A treatment plan was created by using the KBP model, which was compared with the submitted plan for each organ-at-risk (OAR) dose constraint. A report comparing each plan for each OAR constraint was provided to the submitting centre within 24 h of receiving the plan. The centre could then modify the plan based on the KBP report, or continue with the existing plan.

**Results:**

Real-time feedback using KBP was provided in 24/31 cases. Consistent plan quality was in general achieved between KBP and the submitted plan. KBP review resulted in replan and improvement of OAR dosimetry in two patients. All centres indicated that the feedback was a useful QA check of their treatment plan.

**Conclusion:**

KBP for real-time treatment plan review was feasible for 24/31 cases, and demonstrated ability to improve treatment plan quality in two cases. Challenges include integration of KBP feedback into clinical timelines, interpretation of KBP results with respect to clinical trade-offs, and determination of appropriate plan quality improvement criteria.

**Supplementary Information:**

The online version contains supplementary material available at 10.1186/s13014-021-01820-7.

## Introduction

Patient outcomes are dependent on the technical quality of radiation therapy in clinical trials [1–3]. Further, clinical trials in radiation oncology are often used to progress techniques or treatments with demonstrated efficacy in single institution Phase I trials to multi-institution implementations [4, 5]. Appropriate quality assurance (QA) is critical to ensure early positive results can be replicated in a multi-institution setting. QA such as real-time case review prior to treatment can not only reduce the risk profile of novel treatment techniques, but can provide invaluable improvements to treatment quality of each individual patient, in particular for institutions with limited experience with a given technique or anatomical site. Real time QA in clinical trials, however, is resource intensive, requiring substantial effort to de-identify and submit radiation therapy treatment planning data to a central repository and significant investment of time from volunteer reviewers. This is compounded by time constraints, where plan review must be performed efficiently, often in the context of timing with delivery of systemic agents. Typically, dose distributions are often reviewed only to ensure compliance with trial protocol, with limited scope for reviewers to provide input and feedback on whether a treatment plan is optimal for that particular patient.

TROG 15.03 FASTRACK II (ACTRN12615001181594) is a Phase II multi-centre single arm trial with the primary objective of estimating the efficacy of stereotactic ablative body radiation therapy (SABR) for primary renal cell cancer (RCC) (freedom from local progression) [6]. SABR for RCC is technically challenging due to the proximity of multiple organs at risk (OARs) to the target volume, tumour and OAR motion due to respiration, and the often poor image quality with online image guidance in this region [7–9].

Knowledge based planning (KBP) uses previous treatment plans to inform achievable plan quality for new patients [10–12]. KBP estimates achievable OAR dose-volume-histograms (DVHs) for a given patient using a machine learning model. The model has learned achievable dose volume histograms from a library of plans, in which OAR dose depends on the geometric relationship between a target and OAR, beam geometry and prescription dose. KBP has been used to improve the consistency and efficiency of treatment planning in routine practice, to audit treatment plan quality within and external to an institution, and to retrospectively review treatment plan quality in clinical trials [10, 13–21]. In retrospective review of clinical trial data, the potential of KBP to improve individual patient OAR dosimetry has been demonstrated [13, 16, 17, 22–24]. In the current work, we report the use of KBP in real-time QA for the FASTRACK II trial. We used semi-automated KBP review to perform treatment plan quality assessment, in parallel with manual review for a subset of trial participants. This is the first report describing experience of KBP in real-time review of clinical trial plan quality.

## Materials and methods

**Fig. 1:**
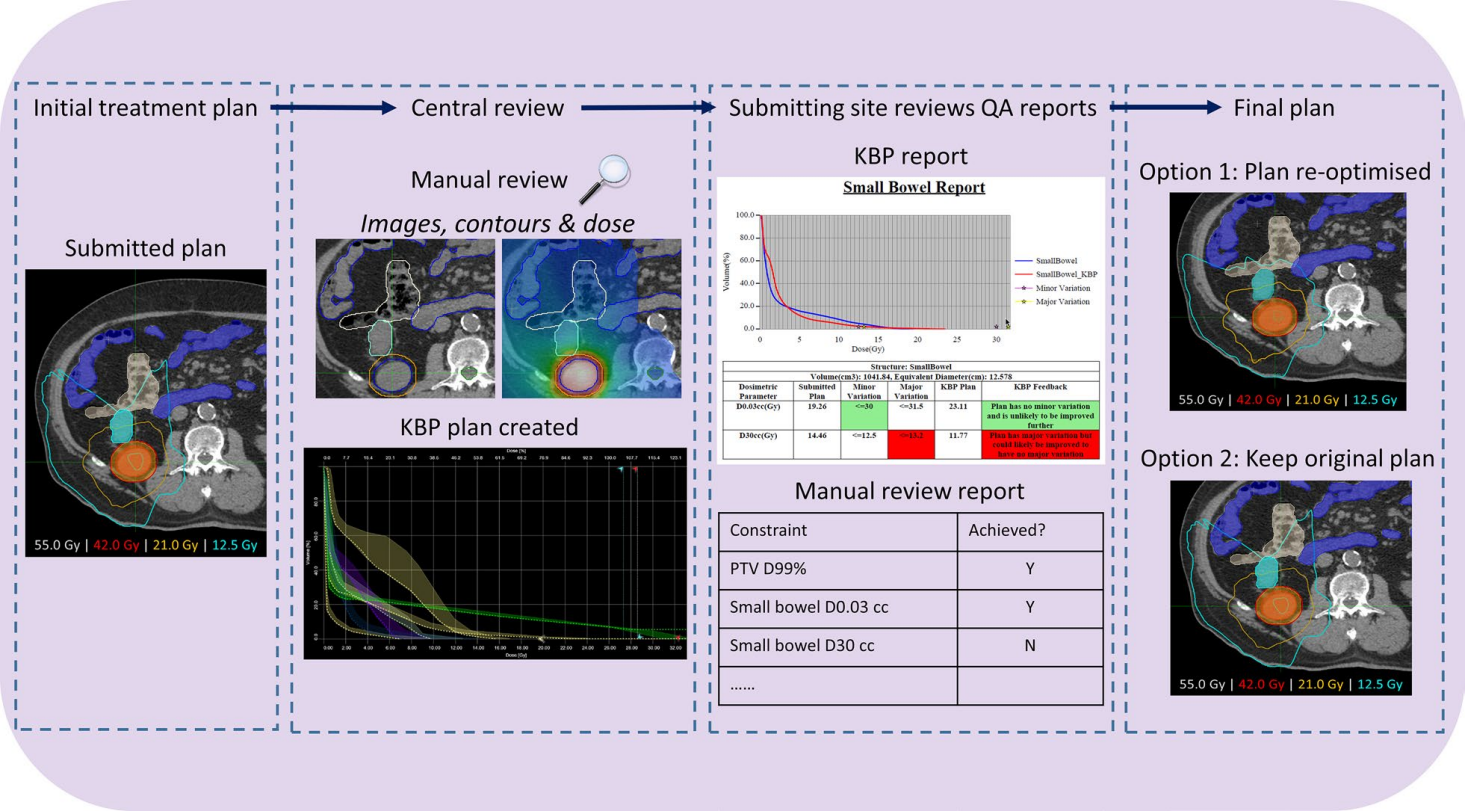
Flowchart for real-time review with KBP. The plan was submitted 7 days prior to treatment date to TROG Central Review. A manual review by a radiation oncologist and medical physicist was performed. TROG RT QA services created a KBP plan for the patient. The submitting institution receives the manual review and a comparison of the KBP plan with the submitted plan, highlighting potential areas of improvement. The submitting centre then has the opportunity to resubmit a new plan

The workflow shown in Fig. 1, adapted from Tol et al. [23], shows the pathway of real-time review used in the current study. For a subset of patients enrolled in the FASTRACK II trial, the submitted treatment planning data (image, structures and treatment beam geometry) was planned using a KBP model. The achievable OAR DVHs with the KBP model was compared with those from the submitted plan, and feedback was provided to the submitting institution, along with the feedback from manual reviewers. The institution could then update the plan, or continue with the existing plan.

### Model generation

In the FASTRACK II trial, participants receive a single fraction of 26 Gy for tumours smaller than 4 cm, or 42 Gy in three fractions for larger tumours. A sequential cohort of 53 previously treated primary kidney cancer patients were used as the training set for the KBP model. These had been treated at a single institution either as part of a prospective Phase I trial or off-trial following the FASTRACK II protocol (23 patients treated with single fraction, 30 treated with three fractions). These patients were selected as they represented the expected cohort that would be recruited in the FASTRACK II trial. This technique was previously demonstrated to be safe with a signal for efficacy [9, 25]. All patients were planned and treated with 3DCRT, using 7–9 beams typically including 1–2 non-coplanar beams. The plans were added into an initial KBP model (RapidPlan, v13.6, Varian Medical Systems), and a series of objectives were created using this KBP model (Additional file 1: Supplementary Table 1). It was expected the majority of plans in the present trial would be treated with a Volumetric Modulated Arc Therapy (VMAT) technique, therefore all patients in this cohort were optimised with a class solution VMAT plan created with two ipsilateral arcs spanning 210° from the posterior-anterior direction around to 30° past midline. The plans were optimised, and refinements were made to objectives to meet the FASTRACK II protocol requirements. In particular, the priority of maximum dose objectives to organs at risk were adjusted to ensure the plans met constraints, and further reduction of mid-range OAR doses was achieved through use of the maximum generalised Equivalent Uniform Dose (gEUD) objective. A new KBP model was created with the final plans. Due insufficient numbers to create two separate models, we created one model for both fractionations. For most OAR constraints, the two fractionations have equivalent OAR constraints as a percentage of the prescription dose. Where this was not the case, we set the objectives to meet the lower constraint of the two fractionations. Validation is described in the Additional file 1: supplementary material.

### Manual review

Real-time review was performed for all patients treated on the trial, and involved review of the CT imaging used for treatment planning, including relevant 4DCT phases, diagnostic imaging used for contouring, structure sets, treatment plans and dose distributions. The plan was submitted ideally seven days prior to treatment start date. The contouring and dose distributions were reviewed by an independent radiation oncologist, and the treatment plan, dose distributions were reviewed by a medical physicist. The review ensured adherence to protocol for contouring, treatment planning parameters, motion management and target and OAR dosimetry. Feedback was provided to the site via a PDF case report with itemised violation criteria, and the site had the opportunity to correct any violations prior to treatment delivery. Subsequent revisions underwent the same review as an initial treatment plan.

### KBP review

KBP review was performed centrally by TROG Cancer Research RTQA Services. In the Eclipse treatment planning system (v13.6, Varian Medical Systems), a generic linac model was created using representative beam data and machine characteristics for each linac type expected to be used in the trial. Only C-arm linear accelerators were used in this trial, and included Varian TrueBeam, Varian TrueBeam STx, Elekta VersaHD and Elekta Synergy linacs. For each patient, a plan was created in Eclipse using the same beam angles and a generic linac model matching that submitted by the centre. The KBP model was applied and the plan was run through one full optimisation without user interaction. An application programing interface (API) script then generated a PDF report which was sent to the site within 24 h of receiving the plan.

For each of the OAR constraints, the KBP generated plan was compared with the submitted plan; the submitted plan value and the KBP plan values were presented, along with the minor and major constraints. Possible improvements were flagged in the report if the submitted plan violated the trial constraint and the KBP plan met the constraint, or if the KBP plan OAR dose was less than 90% of the submitted plan dose. The individual centre then had the opportunity to revise the plan, or continue with the submitted plan, based on their clinical judgement. No plan alterations were mandated. The centre was then invited to provide feedback on the process, using the form presented as Additional file 2: Supplementary Table 2.

For all patients treated in this phase of the FASTRACK II trial, the output of the API script was collected which included the target and OAR metrics for the submitted and KBP plans. Comparison was performed per patient and per metric based on classifications in the report as described above.

## Results

The last 31 patients in the trial were part of the real-time KBP phase, out of 70 patients recruited for the trial. Plans were from 8 centres and included IMRT (1), VMAT (28), dynamic conformal arc (1) and 3D conformal (1) from both Varian and Elekta linacs, and Pinnacle, Eclipse and Monaco treatment planning systems. Only 10/31 cases were provided with at least 7 days prior to radiotherapy start date; real-time reports were however provided in 24/31 cases. Six weren’t provided due to insufficient time between plan submission and treatment for QA staff to provide a report, and one was not provided due to technical issues with the KBP report generation (Additional file 3: Supplementary Table 3). Resubmission of the plan was performed in 10/24 plans. The reason for resubmission was contouring violations in five cases, OAR dosimetry in four cases and insufficient motion management in one case.

### Target dosimetry

The PTV near-minimum and near-maximum doses were similar between the submitted and KBP plans. In the 26 Gy cohort, the PTV_2600 D99% was at or above prescription dose for 13/15 submitted plans, and 11/15 KBP plans, but the D95% was at or above prescription dose for all submitted and KBP plans. In the 42 Gy cohort, the PTV_4200 D99% was at or above prescription dose for 10/16 submitted plans, and 3/16 KBP plans, but the D95% was at or above prescription dose for 15/16 submitted and KBP plans. In one patient the large bowel was adjacent to the target and the PTV coverage was compromised to ensure large bowel constraints were met.

### OAR dosimetry

**Fig. 2:**
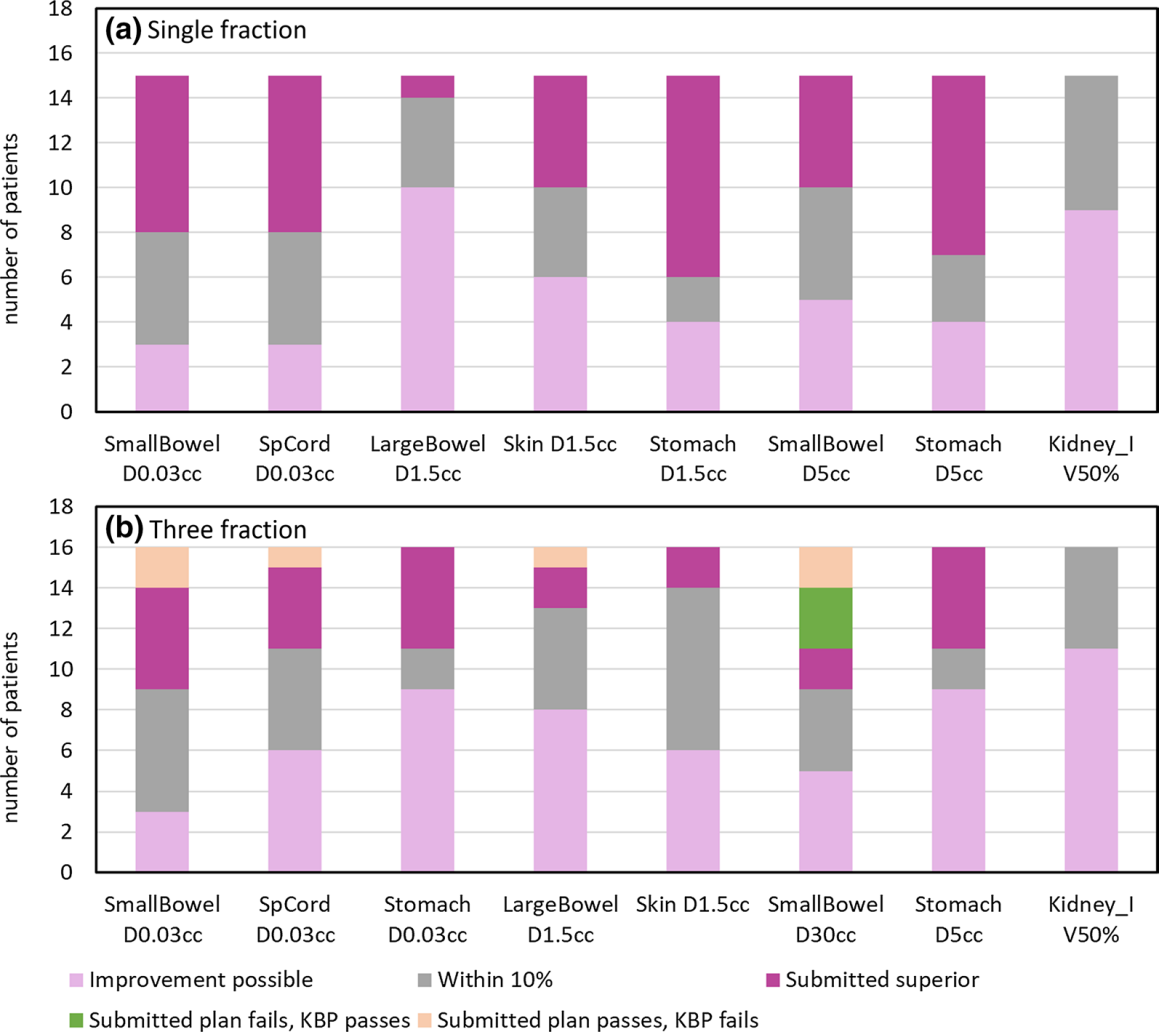
Classification of the KBP result for each OAR metric for **a** single fraction and **b** three fraction schedules. OAR metrics are ordered from maximum dose constraints to volumetric constraints. Improvement possible = KBP was < 90% of submitted plan value; Within 10% = KBP was within 10% of the submitted plan value; Submitted superior = KBP was > 110% of submitted plan value; Submitted plan fails, KBP passes = Submitted plan did not meet constraint, but KBP did; Submitted plan passes, KBP fails = Submitted plan met constraint, but KBP did not. Note the constraint for Kidney_I V50% was ALARA, therefore a plan could not fail this metric

**Fig. 3:**
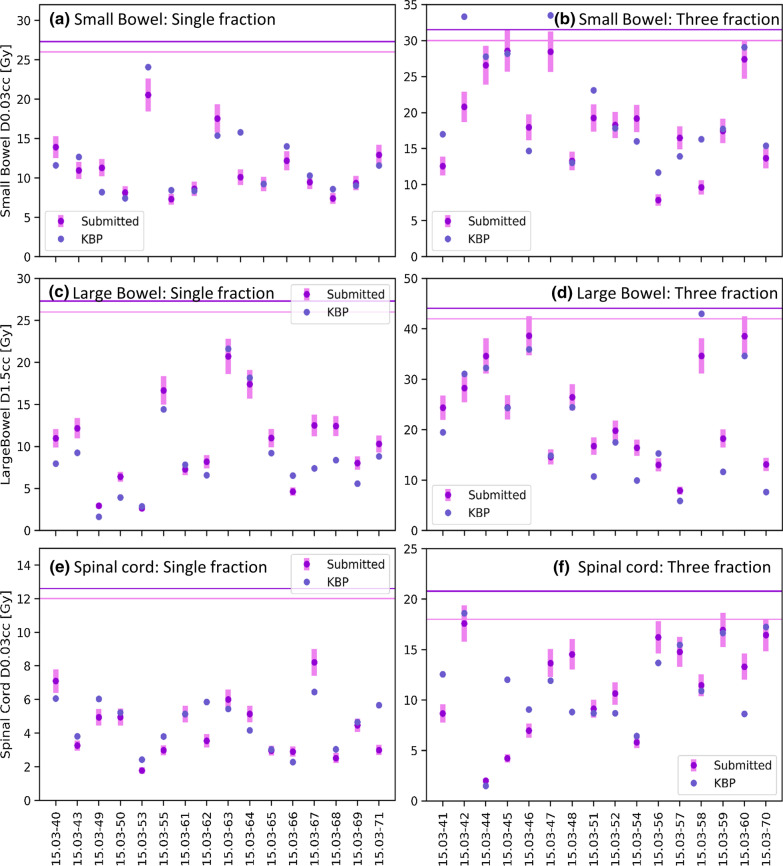
Near-maximum doses for the small and large bowel and spinal cord, for both single and three fraction approaches. The error bars on the submitted plans represent ± 10% of the submitted value, which was used as threshold for suggesting plan improvement. The minor and major violation levels are indicated in the two horizontal lines

**Fig. 4:**
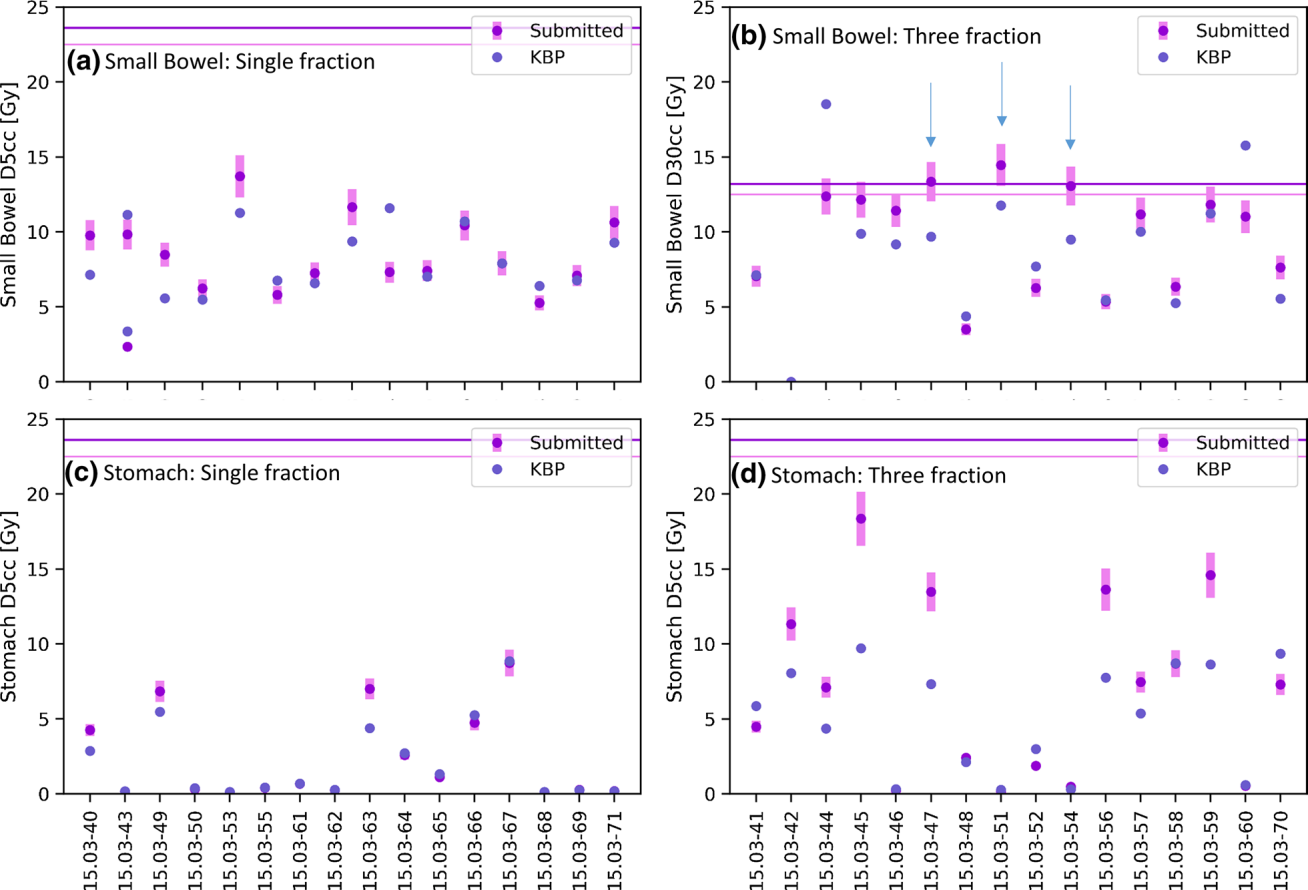
Volume constraints for the small bowel and stomach for the single and three fraction schedules. The error bars on the submitted plans represent ± 10% of the submitted value, which was used as threshold for suggesting plan improvement. The minor and major violation levels are indicated in the two horizontal lines. The arrows on (**b**) indicate where the submitted plan exceeded constraint but KBP did not

Figure 2 classifies the KBP result for each OAR constraint. Figure 3 shows the near-maximum doses for the small bowel, large bowel and spinal cord for both fractionation regimes, and Fig. 4 shows the mid-range DVH metrics for the stomach and small bowel. The near-maximum doses and the DVH constraints were however well below constraints for all plans. There was a relatively even split between KBP being within 10%, or either KBP or the submitted being superior by more than 10%. Improvement was suggested most often for the large bowel D1.5 cc, and Kidney_I V50%. In the three fraction schedule, the near-maximum and mid-range DVH metrics were close to constraint for many patients (Figs. 3, 4). As a result, there were five instances where the submitted plan met the constraint but the KBP plan did not; three were near-maximum doses (small bowel, large bowel and spinal cord) and two were small bowel D30cc. For the small bowel D30cc, there were three submitted plans that did not meet constraints, but the KBP plan did (Fig. 4).

**Fig. 5:**
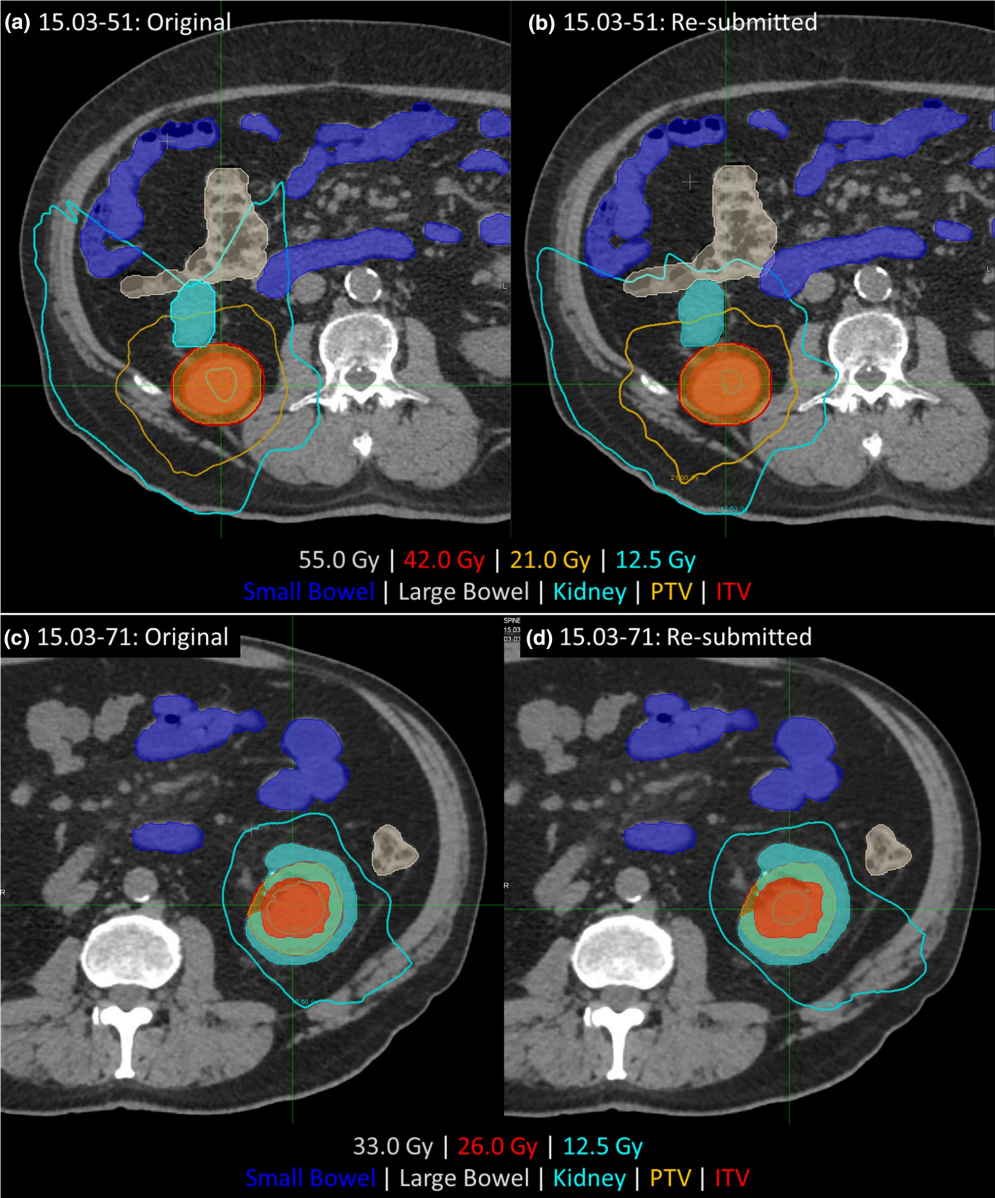
Case 15.03-051 (top row), showing improvements in small bowel and large bowel dose after KBP and manual review both indicated improvements could be made at the anterior dose fall off and (bottom row) Case 15.03-71 showing subtle improvements in the 12.5 Gy isodose line relative to the small bowel

The small bowel D30cc violation was detected by the reviewer for Patient #51 (see Additional files 4, 5: supplementary reports), with the KBP suggesting this constraint could be met. Figure 5a shows the initial plan, with intermediate isodose lines extending across the bowel structures in the submitted plan. The replan (Fig. 5b) shows significant improvement in the intermediate dose spillage, and all constraints met. Patient #54 showed improvement was possible such that the small bowel D30cc constraint was met, but the centre elected to not replan. In this case, objective functions for the small bowel were only used for the near-maximum doses, not the intermediate doses.

There were three plans that were resubmitted based on dosimetric review. For #46, the reviewers recommended reducing target coverage to keep the prescription isodose line off the large bowel. The KBP showed minor improvement, but due to its proximity to the PTV, minimal gains could be made. For patient #60 the reviewers indicated there was a high risk of bowel constraints being exceeded if bowel loops were in a different position at time of treatment. The KBP plan had a similar dose distribution to the submitted plan. In #71, small and large bowel doses were within constraints, but the KBP was more than 10% less than the submitted plan. The site elected to replan this case, in the process reducing small and large bowel dose (Fig. 5c, d), with the aim of improving robustness to bowel position variation at time of treatment.

Feedback was provided by the treating centre for five of 30 participants, and is provided in Additional file 2: Supplementary Table 2. One centre (two patients) indicated the plans could be improved based on the KBP report, however due to time constraints they did not replan. Another centre (two patients) indicated they preferred to retain their chosen clinical trade-offs prioritising one OAR over another. Despite no changes being made in the subset of plans for which feedback was provided, in each case the centre indicated that KBP based QA was a valuable tool for plan quality improvement in clinical trials. Specifically, the participating centres who provided feedback thought analysis of plan quality was useful for their own practice, and for the overall quality of the plans in the trial.

## Discussion

Maintaining quality in clinical trials is important yet resource-intensive. Use of KBP in real-time plan review aims to rapidly identify dosimetric outliers that could be resolved prior to treatment, and provide guidance on optimisation to improve both compliant and non-compliant plans. For clinical trials of new indications or technology, KBP has two key roles. Firstly, this trial was aimed at measuring safety and efficacy of SABR for primary renal cell carcinoma. This was based on a single institution Phase 1 trial which demonstrated this was safe and effective, therefore ideally the treatment planning quality from this Phase 1 trial is carried on to the multi-centre Phase II trial. In this aspect, ensuring the KBP model was at least as good as that used in the Phase I trial maintains this safety, through rapid identification of violations that could be resolved. Secondly, for centres who have little or no experience with kidney SABR, KBP provides a useful reassurance that they are achieving sufficient and consistent plan quality in each of their patients. Plan quality was very consistent between KBP and submitted plans, despite variations in experience, and available software and hardware.

KBP has been used for retrospective review of treatment plan quality in head and neck [16, 22, 23], lung [13] and prostate cancer [24], and vertebral SABR [17]. In all cases, plans performed with KBP were shown to improve the consistency of treatment plan quality, and result in reduced doses to OARs. KBP real-time review processes have been developed for NRG-HN001, facilitated by the Imaging and Radiation Oncology Core [16], and in NRG-GY006 [10, 26]. To our knowledge, this is the first report of prospective use of KBP for real-time review in clinical trial QA. We have demonstrated feasibility of the process, and in two cases in this trial, replanning was performed directly as a result of KBP feedback. In particular, in this trial we had both manual review and KBP based review of the plans; manual reviewers were able to determine whether plans exceeded constraints, but the KBP provided added value by demonstrating that these constraints could in fact be met. The demonstrated improvement in treatment plan quality has the potential to result in improved clinical outcomes for these patients.

We don’t expect that KBP results in superior or equivalent plans in all cases, however we do expect to identify plans with inferior plan quality to the plans upon which the KBP model is based. There were instances where KBP resulted in inferior dosimetry to the submitted plan, in particular where the near-maximum dose in both submitted and KBP plans was close to constraints. High near-maximum dose was due to OAR-target proximity, and manual fine-tuning is likely required, which wasn’t part of the KBP plan generation process. This trade-off mainly affects the near-maximum of the adjacent OAR(s), and the PTV coverage percentage, rather than the intermediate OAR doses. Therefore there may be limited utility in providing feedback based on near-maximum OAR doses and target coverage from KBP-derived plans, with the benefit of KBP mainly arising from ensuring intermediate OAR doses are appropriately optimised. For compliant plans, improvements identified by KBP were in intermediate doses of bowel and ipsilateral kidney, which likely reflects a number of scenarios. Firstly in SABR planning there may not be as much emphasis placed on reducing low and intermediate dose wash in OARs. Secondly, the KBP plan reflects trade-offs between OARs that exist in the model plans, which might be different to trade-offs made by the submitting institution. Lastly, more uniform dose fall-off outside the target may have been preferred over sharp fall off at specific bowel loops. Providing images of dose distributions may assist in interpretation of the KBP plan and help in these scenarios, as recommended by one centre who provided feedback. A further limitation of our approach was to use a single KBP model for both fractionations. Although the relative OAR constraints as a percentage of the prescription dose was very similar in most OARs, the constraints were typically more restrictive for the three fraction regime. Model objectives were set to meet the three fraction constraints, but further improvement may be achievable by having fraction-specific models.

There are a number of challenges with interpretation of KBP generated estimates for deciding whether to re-optimise a plan. Firstly, we provided per-structure recommendations. Certain OARs may have been prioritised over others at the discretion of the treating team, and this is not taken into account by the KBP-based recommendations. Secondly, we elected to suggest OAR sparing when the KBP achieved metric was at least 10% lower than that in the submitted plan, as opposed to any gain. This was performed in attempt to ensure suggestion was made when clinically meaningful. Giaddui et al. suggest 5% is suitable for head and neck cancer, based on analysis of potential gains and link to calibration dosimetry. Ideally improvement is suggested based on normal tissue complication probability (NTCP) [24]. That is, if the level of OAR dose is where the NTCP curve is flat, a large change in OAR dose would be required to determine a clinically relevant gain, and where the NTCP curve is steep, a small change in dose would lead to a large gain. In abdominal SABR however, there is a lack of dose response data for relevant OARs, and constraints are mainly derived from serial structure toxicities. Lastly, it may also be argued that plan modulation metrics should also be considered as part of real-time review, both to ensure the submitted plan is not over-modulated, and to ensure that any KBP suggested improvements are not at the expense of over-modulation which may impact deliverability.

Short turnaround time impacts feasibility of KBP QA, as indicated by the lack of real-time KBP review provided for six cases and the feedback from participating centres. The 7 day criteria was an arbitrary timeline selected to allow manual handling of datasets and central transfer with a sufficient time period to enable the start date of radiotherapy within a clinical trial timeframe. An alternative approach is provision of a KBP model to participating centres at trial inception, facilitating estimated DVHs or objectives at time of rather than after treatment planning, such that that the KBP report is incorporated within the final plan check process. This would not necessitate such long lead-times as was designed in this trial. Further, improvements in plan quality must be balanced with additional resources required with plan modification. Further, it is proposed that a KBP model or OAR DVHs are summarised from the treatment plans used for treatment, to facilitate replication of trial results in the real-world setting [27].

## Conclusion

In this prospective trial evaluating safety and efficacy of SABR for kidney cancer, we have demonstrated KBP for real-time review of treatment plan quality can be feasible. Challenges were faced with timelines between plan submission, provision of the KBP report, and treatment start date. KBP demonstrated utility in highlighting plans that exceeded tolerance and may be improved through further optimisation. There are however some potential improvements to this process, such as provision of KBP models to participating sites at the start of the trial, and improved quality improvement thresholds. Such improvements may result in substantial potential for automation of real-time review adding further emphasis on plan quality.

## Supplementary Information

**Additional file 1: Supplementary Table 1.** Optimisation objectives used for KBP plan generation.** Supplementary Figure 1**: Comparison of the submitted and KBP plan metrics for 11 patients out of the first 40 submitted to the trial.

**Additional file 2.** TROG 15.03 FASTRACKII KBP Feedback Report– Patient Specific Questionnaire.

**Additional file 3.** Details of case submission timelines and outcome of KBP review.

**Additional file 4.** Radiotherapy Pre-treatment case report: Case #51.

**Additional file 5.** KBP Quality Feedback Report: Case #51.

## Data Availability

The datasets used and/or analysed during the current study are available from the corresponding author on reasonable request.

## Abbreviations

API: Application programming interface
DVH: Dose volume histogram
IMRT: Intensity modulated arc therapy
KBP: Knowledge based planning
NTCP: Normal tissue complication probability
OAR: Organ at risk
PTV: Planning target volume
QA: Quality assurance
RCC: Renal cell cancer
SABR: Stereotactic ablative body radiotherapy
VMAT: Volumetric modulated arc therapy
