# Supplementary material for: Personalising treatment plan quality review with knowledge-based planning in the TROG 15.03 trial for stereotactic ablative body radiotherapy in primary kidney cancer
Source: Radiat Oncol. 2021 Aug 3;16:142. doi: 10.1186/s13014-021-01820-7 (PMC8330099; doi:10.1186/s13014-021-01820-7)
Supplement: Supplementary file 1 — Additional file 1: Supplementary Table 1. Optimisation objectives used for KBP plan generation. Supplementary Figure 1: Comparison of the submitted and KBP plan metrics for 11 patients out of the first 40 submitted to the trial. [file 13014_2021_1820_MOESM1_ESM.docx]

## Supplementary Data

Supplementary Table 1: Optimisation objectives

| **Structure** | **Objective** | **Volume (%)** | **Dose (%)** | **Priority** |
| --- | --- | --- | --- | --- |
| ITV | Upper | 0 | 130 | 100 |
| ITV | Lower | 2 | 125 | 100 |
| ITV | Lower | 100 | 110 | 100 |
| PTV-ITV | Upper | 0 | 110 | 100 |
| PTV-ITV | Lower | 100 | 100 | 120 |
| Kidney_I | Line |  |  | Gen. |
| Kidney_C | Line |  |  | Gen. |
| SmallBowel_prox* | Line |  |  | Gen. |
| SmallBowel_prox* | Upper | 0 | 70 | 100 |
| LargeBowel | Line |  |  | Gen. |
| LargeBowel | Upper | 0 | 100 | 100 |
| Skin | Line |  |  | Gen. |
| Skin | Upper | 0 | 70 | 100 |
| Liver | Line |  |  | Gen. |
| Stomach | Line |  |  | Gen. |
| Stomach | Upper | 0 | 70 | 100 |
| SpCord | Line |  |  | Gen. |
| SpCord | Upper | 0 | 40 | 100 |

*SmallBowel_prox was the small bowel within 5 cm of the PTV and was used to ensure a consistent optimisation volume between patients. There was variation between patients in how much small bowel was contoured at a distance from the PTV.

**Validation**

For validation, the model was applied to a random selection of 11 of the first 40 submitted plans in the FASTRACK II trial. The KBP was run through a single optimisation without user interaction. The resultant KBP derived plans were compared with those from the submitted plan. The results are provided in Supplementary Figure 1, and indicate consistent achievement of protocol constraints with KBP, and largely consistent target and OAR dosimetry between the submitted plans and the KBP plans.


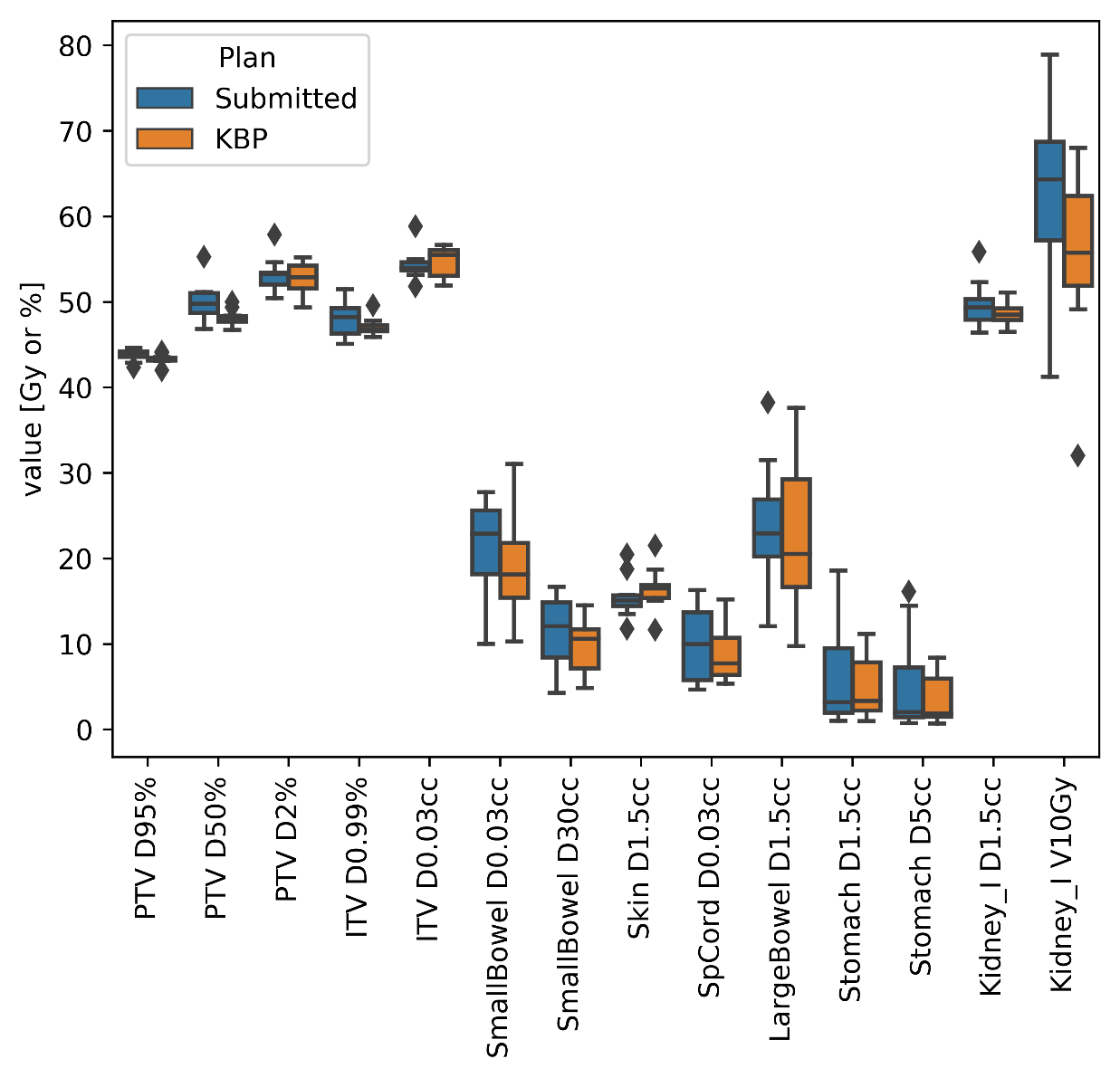


Supplementary Figure 1: Comparison of the submitted and KBP plan metrics for 11 patients out of the first 40 submitted to the trial.
