# Supplementary material for: Personalising treatment plan quality review with knowledge-based planning in the TROG 15.03 trial for stereotactic ablative body radiotherapy in primary kidney cancer
Source: Radiat Oncol. 2021 Aug 3;16:142. doi: 10.1186/s13014-021-01820-7 (PMC8330099; doi:10.1186/s13014-021-01820-7)
Supplement: Supplementary file 4 — Additional file 4. Radiotherapy Pre-treatment case report: Case #51. [file 13014_2021_1820_MOESM4_ESM.pdf]

# Knowledge-based Plan Quality Feedback Report for FASTRACK-RENAL

**Patient Identifier: 15.03_1503-051**
**Report prepared on: 7/8/2019 12:00:00 AM**

**Dosimetric Analysis Summary:**

| Structure | Variations | KBP feedback statement |
|---|---|---|
| PTV | All dose metrics are acceptable | Dose metrics are unlikely to be improved further |
| ITV | All dose metrics are acceptable | Dose metrics are unlikely to be improved further |
| SmallBowel | Dose metrics have major variations | Plan could be improved to be within acceptable variation |
| Skin | All dose metrics are acceptable | Some dose metrics could likely be improved |
| SpCord | All dose metrics are acceptable | Dose metrics are unlikely to be improved further |
| LargeBowel | All dose metrics are acceptable | Some dose metrics could likely be improved |
| Stomach | All dose metrics are acceptable | Dose metrics are unlikely to be improved further |
| Kidney_I | All dose metrics are acceptable | Dose metrics are unlikely to be improved further |
| Kidney_C | All dose metrics are acceptable | Dose metrics are unlikely to be improved further |
| Liver | All dose metrics are acceptable | Dose metrics are unlikely to be improved further |

Please review the subsequent pages of this report for individual structure DVH analysis and comparison to the knowledge-based planning reference for this patient. These should be interpreted primarily as verification that the plan quality submitted is similar to how previous patients treated to this protocol have been planned. This process is aimed at reducing outliers in plan quality. Differences between KBP and the submitted plans are presented; for OARs where KBP is greater than 10% lower than the submitted plan, this is highlighted. Where differences exist between KBP and the submitted plan, it is entirely up to your clinical judgement to determine whether the improvement would be clinically beneficial for the patient. This includes taking into account trade-offs that may have been made between different organs which may have led to differences between the KBP and submitted plans. Details of how the model has been built, frequently asked questions and references regarding the basis for KBP can be found at the end of this report.

# Knowledge-based Plan Quality Feedback Report for FASTRACK-RENAL

**Patient Identifier: 15.03_1503-051**
**Report prepared on: 7/8/2019 12:00:00 AM**

# PTV Report

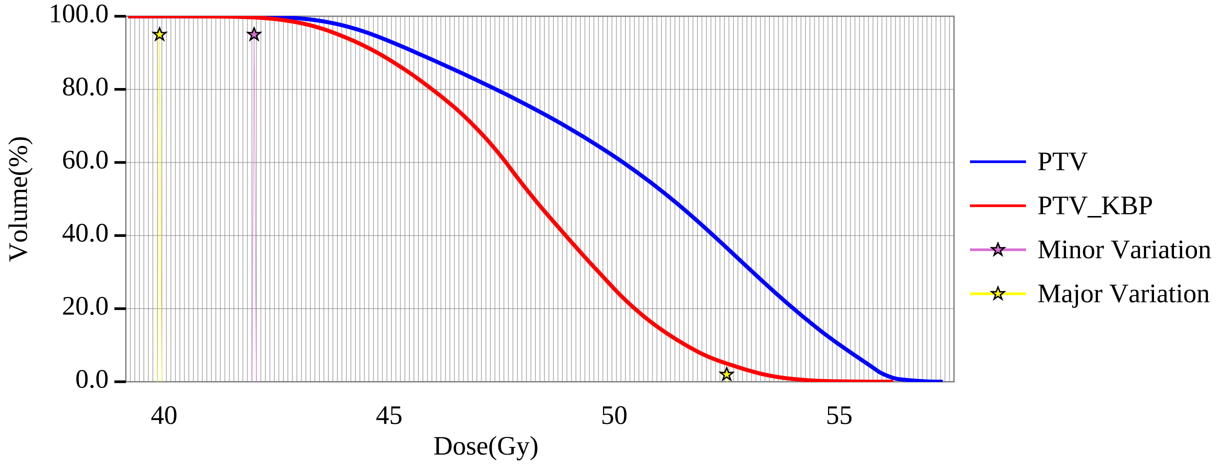


| Structure: PTV | | | | | |
|---|---|---|---|---|---|
| Volume(cm3): 127.15, Equivalent Diameter(cm): 6.24 | | | | | |
| **Dosimetric Parameter** | **Submitted Plan** | **Minor Variation** | **Major Variation** | **KBP Plan** | **KBP Feedback** |
| D95%(Gy) | 44.63 | >=41.958 | >=39.9 | 43.87 | Plan has no minor variation and is unlikely to be improved further |
| D99%(Gy) | 43.32 | - | - | 42.62 | |
| DMEAN(Gy) | 50.75 | - | - | 48.22 | |
| DMAX(Gy) | 57.21 | >=60.06 | >=52.5 | 56.18 | Plan has minor variation but is unlikely to be improved further |
| D50%(Gy) | 51.28 | - | - | 48.22 | |
| D2%(Gy) | 56 | - | - | 53.33 | |
| D0.03cc(Gy) | 57.07 | - | >=52.5 | 55.54 | Plan has no minor variation and is unlikely to be improved further |

# Knowledge-based Plan Quality Feedback Report for FASTRACK-RENAL

**Patient Identifier: 15.03_1503-051**
**Report prepared on: 7/8/2019 12:00:00 AM**

# ITV Report

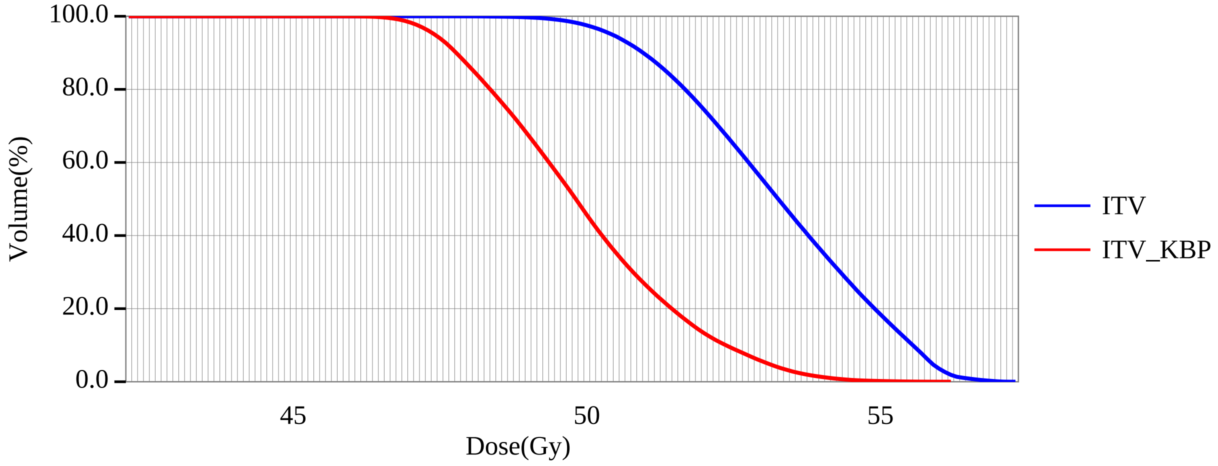


| Structure: ITV | | | | | |
|---|---|---|---|---|---|
| Volume(cm3): 70.49, Equivalent Diameter(cm): 5.13 | | | | | |
| **Dosimetric Parameter** | **Submitted Plan** | **Minor Variation** | **Major Variation** | **KBP Plan** | **KBP Feedback** |
| D99%(Gy) | 49.52 | - | - | 46.83 | |
| D0.03cc(Gy) | 57.07 | - | - | 55.54 | |

# Knowledge-based Plan Quality Feedback Report for FASTRACK-RENAL

**Patient Identifier: 15.03_1503-051**

**Report prepared on: 7/8/2019 12:00:00 AM**

# Small Bowel Report

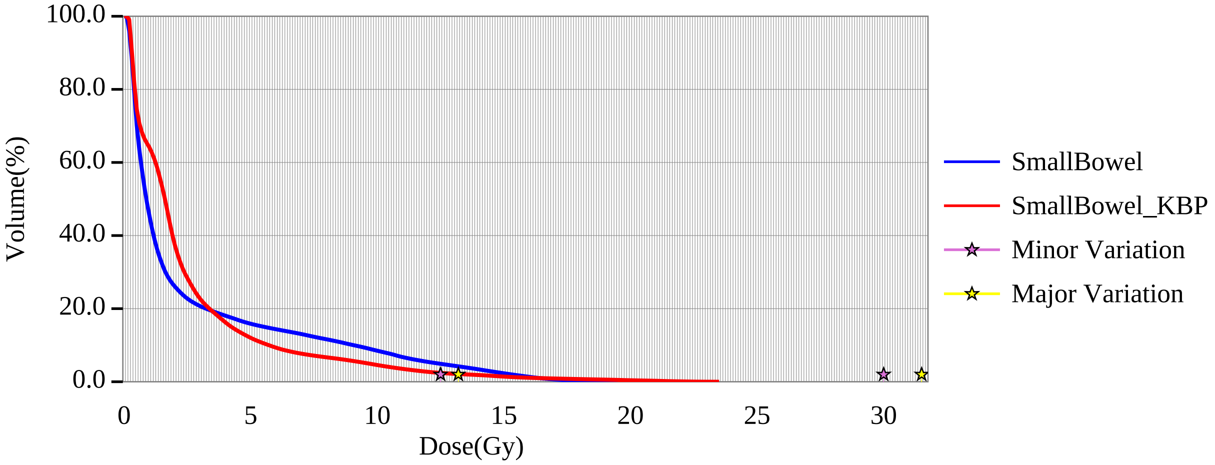


| Structure: SmallBowel | | | | | |
|---|---|---|---|---|---|
| Volume(cm3): 1041.84, Equivalent Diameter(cm): 12.578 | | | | | |
| **Dosimetric Parameter** | **Submitted Plan** | **Minor Variation** | **Major Variation** | **KBP Plan** | **KBP Feedback** |
| **D0.03cc(Gy)** | **19.26** | **<=30** | **<=31.5** | **23.11** | **Plan has no minor variation and is unlikely to be improved further** |
| **D30cc(Gy)** | **14.46** | **<=12.5** | **<=13.2** | **11.77** | **Plan has major variation but could likely be improved to have no major variation** |

# Knowledge-based Plan Quality Feedback Report for FASTRACK-RENAL

**Patient Identifier: 15.03_1503-051**
**Report prepared on: 7/8/2019 12:00:00 AM**

# Skin Report

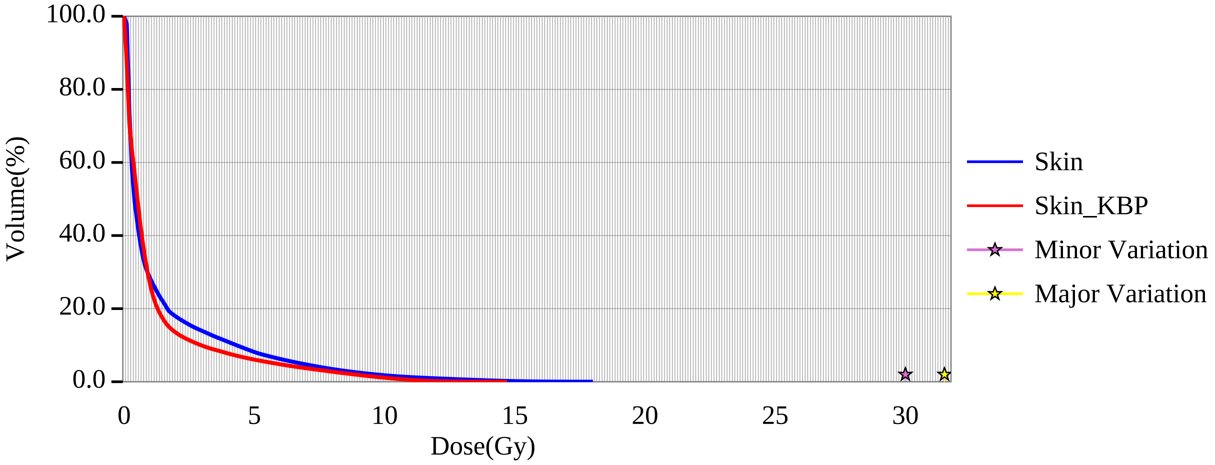


| Structure: Skin | | | | | |
|---|---|---|---|---|---|
| Volume(cm3): 630.84, Equivalent Diameter(cm): 10.641 | | | | | |
| **Dosimetric Parameter** | **Submitted Plan** | **Minor Variation** | **Major Variation** | **KBP Plan** | **KBP Feedback** |
| **D1.5cc(Gy)** | **14.59** | **<=30** | **<=31.5** | **11.78** | **Plan has no minor variation but could likely be improved further** |

# Knowledge-based Plan Quality Feedback Report for FASTRACK-RENAL

**Patient Identifier: 15.03_1503-051**

**Report prepared on: 7/8/2019 12:00:00 AM**

# Spinal Canal Report

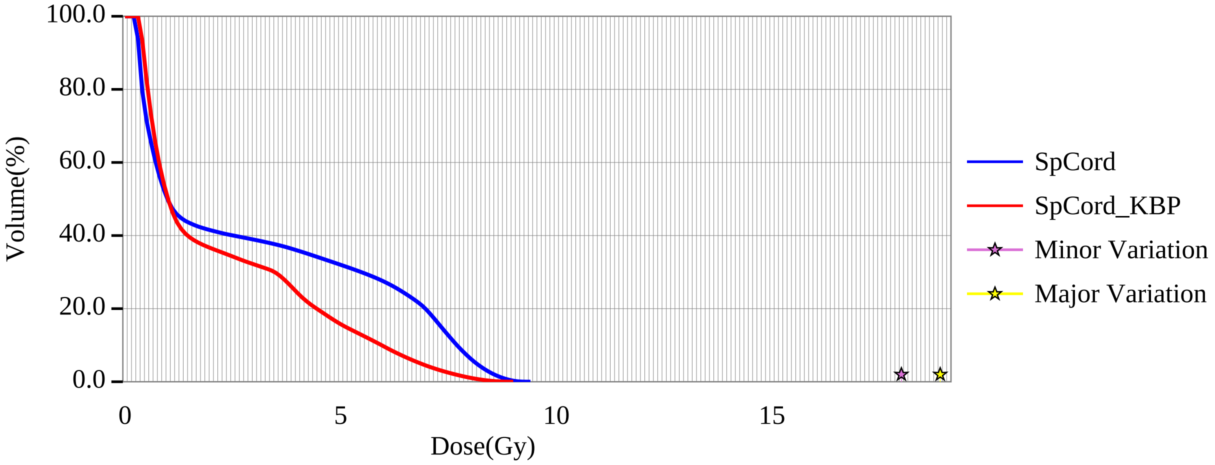


| Structure: SpCord | | | | | |
|---|---|---|---|---|---|
| Volume(cm3): 40.36, Equivalent Diameter(cm): 4.256 | | | | | |
| **Dosimetric Parameter** | **Submitted Plan** | **Minor Variation** | **Major Variation** | **KBP Plan** | **KBP Feedback** |
| D0.03cc(Gy) | 9.13 | <=18 | <=18.9 | 8.69 | Plan has no minor variation and is unlikely to be improved further |

# Knowledge-based Plan Quality Feedback Report for FASTRACK-RENAL

**Patient Identifier: 15.03_1503-051**

**Report prepared on: 7/8/2019 12:00:00 AM**

# Large Bowel Report

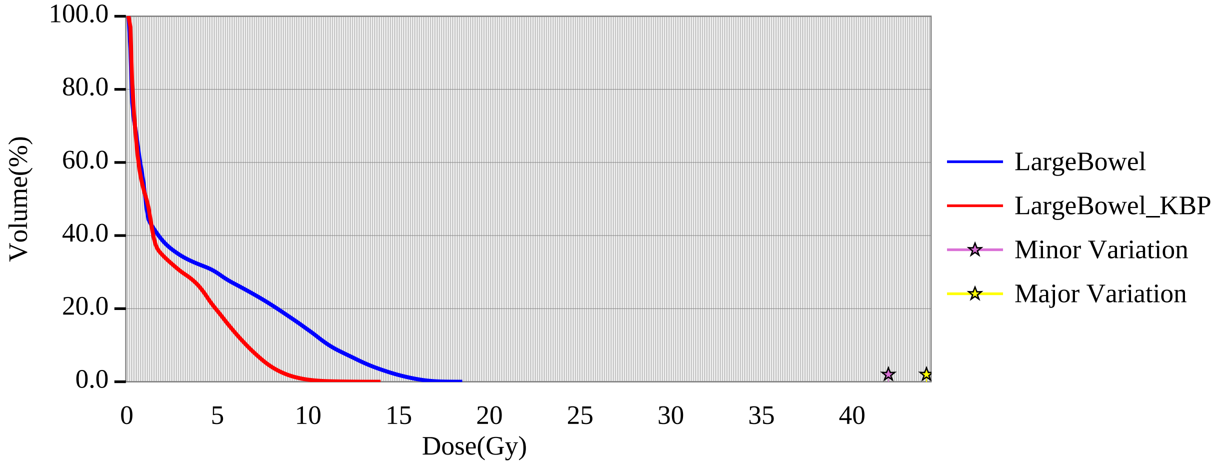


| Structure: LargeBowel | | | | | |
|---|---|---|---|---|---|
| Volume(cm3): 672.9, Equivalent Diameter(cm): 10.872 | | | | | |
| **Dosimetric Parameter** | **Submitted Plan** | **Minor Variation** | **Major Variation** | **KBP Plan** | **KBP Feedback** |
| **D1.5cc(Gy)** | **16.75** | **<=42** | **<=44.1** | **10.74** | **Plan has no minor variation but could likely be improved further** |

# Knowledge-based Plan Quality Feedback Report for FASTRACK-RENAL

**Patient Identifier: 15.03_1503-051**
**Report prepared on: 7/8/2019 12:00:00 AM**

# Stomach Report

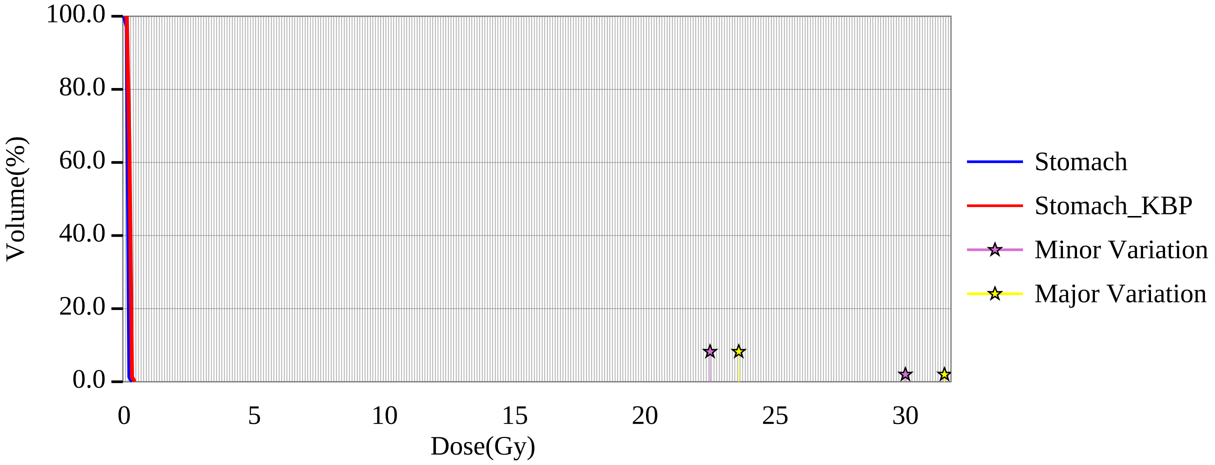


| Structure: Stomach | | | | | |
|---|---|---|---|---|---|
| Volume(cm3): 60.71, Equivalent Diameter(cm): 4.876 | | | | | |
| **Dosimetric Parameter** | **Submitted Plan** | **Minor Variation** | **Major Variation** | **KBP Plan** | **KBP Feedback** |
| **D0.03cc(Gy)** | **0.22** | **<=30** | **<=31.5** | **0.31** | **Plan has no minor variation and is unlikely to be improved further** |
| **D5cc(Gy)** | **0.17** | **<=22.5** | **<=23.6** | **0.28** | **Plan has no minor variation and is unlikely to be improved further** |

# Knowledge-based Plan Quality Feedback Report for FASTRACK-RENAL

**Patient Identifier: 15.03_1503-051**

**Report prepared on: 7/8/2019 12:00:00 AM**

# Ipsilateral Kidney Report

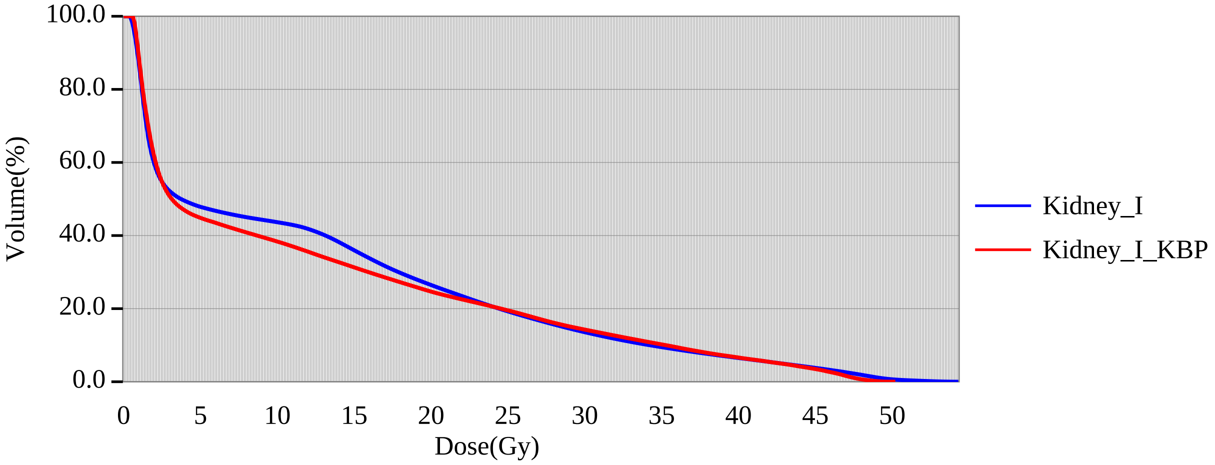


| Structure: Kidney_I | | | | | |
|---|---|---|---|---|---|
| Volume(cm3): 228.28, Equivalent Diameter(cm): 7.583 | | | | | |
| **Dosimetric Parameter** | **Submitted Plan** | **Minor Variation** | **Major Variation** | **KBP Plan** | **KBP Feedback** |
| **D1.5cc(Gy)** | **50** | **-** | **-** | **48** | |
| **V10Gy(%)** | **43.65** | **-** | **-** | **38.35** | |
| **V21Gy(%)** | **24.93** | **-** | **-** | **23.55** | |

# Knowledge-based Plan Quality Feedback Report for FASTRACK-RENAL

**Patient Identifier: 15.03_1503-051**
**Report prepared on: 7/8/2019 12:00:00 AM**

# Contralateral Kidney Report

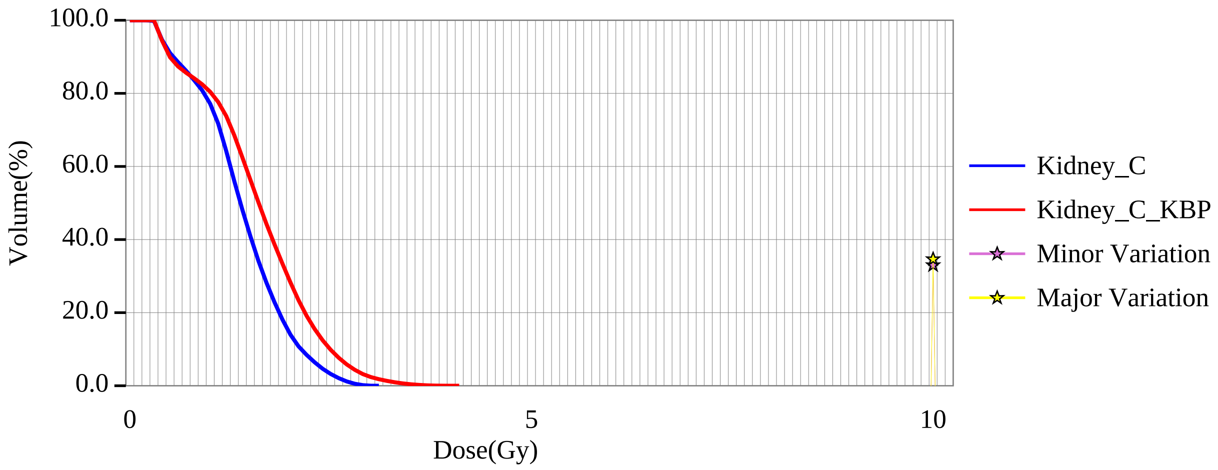


| Structure: Kidney_C | | | | | |
|---|---|---|---|---|---|
| Volume(cm3): 277.34, Equivalent Diameter(cm): 8.091 | | | | | |
| **Dosimetric Parameter** | **Submitted Plan** | **Minor Variation** | **Major Variation** | **KBP Plan** | **KBP Feedback** |
| **V10Gy(%)** | **0** | **<=33** | **<=34.65** | **0** | **Plan has no minor variation and is unlikely to be improved further** |
| **D0.03cc(Gy)** | **2.99** | **-** | **-** | **3.9** | |

# Knowledge-based Plan Quality Feedback Report for FASTRACK-RENAL

**Patient Identifier: 15.03_1503-051**

**Report prepared on: 7/8/2019 12:00:00 AM**

# Liver Report

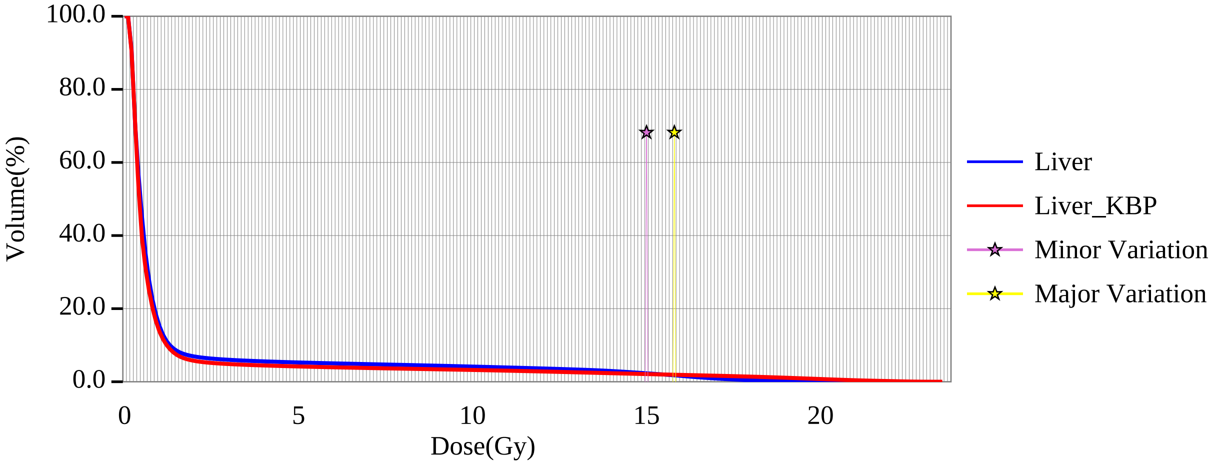


| Structure: Liver | | | | | |
|---|---|---|---|---|---|
| Volume(cm3): 1026.19, Equivalent Diameter(cm): 12.514 | | | | | |
| **Dosimetric Parameter** | **Submitted Plan** | **Minor Variation** | **Major Variation** | **KBP Plan** | **KBP Feedback** |
| **D700cc(Gy)** | **0.32** | **<=15** | **<=15.8** | **0.32** | **Plan has no minor variation and is unlikely to be improved further** |
| **D0.03cc(Gy)** | **20.53** | **-** | **-** | **23.18** | |

# Knowledge-based Plan Quality Feedback Report for FASTRACK-RENAL

**Patient Identifier: 15.03_1503-051**
**Report prepared on: 7/8/2019 12:00:00 AM**

**Objectives used in KBP plan:**

| KBP plan Objectives | | | | | |
|---|---|---|---|---|---|
| Structure | Type | Limit | Volume(%) | Dose(Gy) | Priority |
| PTV-ITV | Point | Lower | 100.00 | 42.00 | 100 |
| PTV-ITV | Point | Upper | 0.00 | 46.20 | 43.147298 |
| SmallBowel_prox | Point | Upper | 0.00 | 25.20 | 66.249627 |
| SmallBowel_prox | Line | Upper | - | - | 66.249627 |
| Stomach | Line | Upper | - | - | 100 |
| SpCord | Point | Upper | 0.00 | 14.70 | 83.828984 |
| SpCord | Line | Upper | - | - | 83.828984 |
| Skin | Point | Upper | 0.00 | 21.00 | 100 |
| Skin | Line | Upper | - | - | 100 |
| Liver | Line | Upper | - | - | 100 |
| LargeBowel | Line | Upper | - | - | 69.583238 |
| Kidney_I | Line | Upper | - | - | 70.457626 |
| Kidney_C | Line | Upper | - | - | 70.883217 |
| ITV | Point | Lower | 100.00 | 46.20 | 100 |
| ITV | Point | Upper | 0.00 | 54.60 | 46.146964 |

# Frequently Asked Questions

1. How is the KBP reference plan generated?

   *Knowledge-based planning is accomplished by collecting samples of previously treated patients and training predictive models that yield accurate predictions of final plan dosimetry based on individual patient anatomy[1-4]. These predictions are then used to inform inverse optimization by automatically generating patient-specific optimization objectives.*

2. What technical parameters were used for the KBP plans?

   *The knowledge-based plans were developed in Varian's RapidPlan using dose-volume estimation models trained with multi-arc volumetric-modulated arc (VMAT) plans on C-arm linear accelerators. The KBP reference plan presented in this report was developed in the same treatment planning system and represents a deliverable plan on a VMAT-capable Varian linear accelerator (15MV photons, 120-leaf Millenium MLCs).*

3. How are the knowledge-based feedback statements generated?

   *The language behind the KBP feedback statements associated with each plan quality metric are obtained from the following table for normal tissue structures:*

| Variable Definitions |
|---|
| Ds = Soft Constraint |
| Dh = Hard Constraint |
| Dplan = Submitted plan value |
| Dkpb = KBP value |

| Scenario | Analysis | KBP scenario | KBP feedback statement |
|---|---|---|---|
| Dplan <= Ds | Meets Soft Constraint | Dplan <= Dkbp | Plan meets soft constraint and is unlikely to be improved further |
| | | Dkbp < Dplan | Plan meets soft constraints but this dose metric could likely be improved further |
| Ds < Dplan <= Dh | Exceeds Soft Constraint | Dplan <= Dkbp | Plan exceeds soft constraint but this dose metric is unlikely to be improved further |
| | | Ds < Dkbp < Dplan | Plan exceeds soft constraint but this dose metric could likely be improved further |
| | | Dkbp <= Ds < Dplan | Plan meets soft constraint but this dose metric could likely be improved to meet soft constraint |
| Dplan > Dh | Exceeds hard Constraint | Dplan <= Dkbp | Plan exceeds hard constraint but this dose metric is unlikely to be improved further |
| | | Dh < Dkbp < Dplan | Plan exceeds hard constraint but this dose could likely be improved further |
| | | Dkbp <= Dh < Dplan | Plan exceeds hard constraint but this dose metric could likely be improved to meeth hard constraint |

*An equivalent table is used for target coverage metrics with the "less than" inequalities converted to "greater than" conditions.*

5. I cannot make my re-plan meet every element of the KBP reference plan, what should I do?

*The KBP plan represents a deliverable plan according to the planning parameters specified in #2. More often than not, highly similar plans should be attainable. However, depending on treatment planning system and/or machine delivery configuration, an exactly equivalent plan might be difficult or even impossible to achieve. Participating sites are encouraged to use their clinical judgment and practical time constraints in regards to the effort put forth to match the KBP reference plans.*

References:


**1.** Zhu X *et al.,* A planning quality evaluation tool for prostate adaptive IMRT based on machine learning. *Medical Physics.* Feb 2011;38(2):719-726.

**2.** Appenzoller LM *et al.,* Predicting dose-volume histograms for organs-at-risk in IMRT planning. *Medical Physics.* 2012;39(12):7446.

**3.** Moore KL *et al.*, Quantifying Unnecessary Normal Tissue Complication Risks due to Suboptimal Planning: A Secondary Study of RTOG 0126. *International Journal of Radiation Oncology* Biology* Physics.* 2015;92(2):228-235.

**4.** Li N *et al.*, Validation of a Knowledge Based Automated Planning System in Cervical Cancer As a Clinical Trial Quality System, Annual Meeting of the American Society for Radiation Oncology, San Antonio, TX (2015)
