# Supplementary material for: Personalising treatment plan quality review with knowledge-based planning in the TROG 15.03 trial for stereotactic ablative body radiotherapy in primary kidney cancer
Source: Radiat Oncol. 2021 Aug 3;16:142. doi: 10.1186/s13014-021-01820-7 (PMC8330099; doi:10.1186/s13014-021-01820-7)
Supplement: Supplementary file 5 — Additional file 5. KBP Quality Feedback Report: Case #51. [file 13014_2021_1820_MOESM5_ESM.pdf]

TROG 15.03 - FASTRACK II - A multicentre phase II clinical trial of stereotactic ablative body radiotherapy for primary kidney cancer.

Quality Assurance Reviews

RADIOTHERAPY PRE-TREATMENT - CASE REPORT

CASE NUMBER: [redacted] 1503-051
PARTICIPATING CENTRE: [redacted]
PRINCIPAL INVESTIGATOR: [redacted]
TREATING CLINICIAN: [redacted]
REVIEW TYPE: Initial

1. Introduction

Prior to radiotherapy treatment commencement, treatment plans for all patients are to be submitted to the TROG QA office for RT QA review.

Investigators were asked to provide copies of source documentation for each case according to QA checklists. The source documentation was reviewed to verify that the case complied with the protocol.
Case reports are provided to the investigator following the completion of each case review.

2. Centre Summary

Review Results: Resubmission Required

3. Radiotherapy Pre-Treatment Review

3.1. Classification of Review Results

Review results are classified according to defined standards for deviations/variations. The categories are assigned as follows:
- Acceptable: no variation or variation within tolerance limits
- Minor/Lesser Deviation: variation that will not have significant impact on the outcome or interpretation of the study but may require follow-up or education to prevent recurrence in subsequent cases or progression to major deviations.
- Major Deviation: variation from protocol-specified procedures that makes the resulting data quesitonable and may affect the interpretation of the endpoints.
- Missing/Inevaluable: review not possible based on the documentation provided.

Please contact the TROG Central Operations Office if you would like a copy of the variables reviewed and variation classifications.
Principal Investigators are welcome to provide additional information and to seek clarification if it is considered that a variation has not been correctly attributed to this case

3.2. Case Result Summary

Categories	Variables Per Case	Acceptable	Minor Variation	Major Variation	Missing / Inevaluable
Dose	17	16	0	1	0
Technique	14	13	0	1	0
**Total**	**31**	**29 (93.5%)**	**0 (0%)**	**2 (6.5%)**	**0 (0%)**

3.3. Case Result Details

Categories	Acceptable	Minor Variation	Major Variation	Missing / Inevaluable
**Dose**				
Was 42Gy Prescribed?	1	0	0	0
Number of fractions	1	0	0	0
Dose per fraction (Gy)	1	0	0	0
PTV (D95%)	1	0	0	0
PTV Dmax (%)	1	0	0	0
PTV CI 100%	1	0	0	0
Spinal Canal Max (0.03cc) Gy	1	0	0	0
Skin Max (1.5cc) Gy	1	0	0	0
Small Bowel max (0.03cc) Gy	1	0	0	0
Small Bowel (D30cc) Gy	0	0	1	0
Small Bowel max dose covering full circumference of bowel wall	1	0	0	0
Large Bowel Max (1.5cc) Gy	1	0	0	0
Stomach Max (0.03cc) Gy	1	0	0	0
Stomach (5cc) Gy	1	0	0	0
Liver (700cc) Gy	1	0	0	0
Contralateral Kidney (V10Gy) %	1	0	0	0
Is max dose located within the ITV?	1	0	0	0
**Technique**				
Maximum Energy (MV)	1	0	0	0
Dose calculation Matrix < or = 0.3cm	1	0	0	0
Inhomogenity correction on?	1	0	0	0
CT scan (please see comment)	1	0	0	0
PTV Contour	1	0	0	0
ITV Contour	1	0	0	0
Liver Contour	1	0	0	0
Spinal Cord Contour	1	0	0	0
Contralateral Kidney Contour	1	0	0	0
Ipsilateral Kidney Contour	1	0	0	0
Small Bowel Contour	1	0	0	0
Large Bowel Contour	1	0	0	0
Stomach Contour	0	0	1	0
Skin Contour	1	0	0	0
**Total**	**29 (93.5%)**	**0 (0%)**	**2 (6.5%)**	**0 (0%)**

3.4. Case Result Comments

Major Variations

2 major variations were recorded for this patient.
-Technique Stomach Contour
-Dose Small Bowel (D30cc) Gy

Minor Variations

No minor variations were recorded for this patient.

Missing/Inevaluable Data

All required data was submitted for review.

4. Trial QA Comments

Reviewers Comments:
Dose grid not covering all structures. Small Bowel D30cc Major Violation.
Stomach not contoured inferiorly.
Resubmission Required

Please contact the Trial Chairperson or TROG Central Operations Office (QA) with any queries, or if you wish to discuss the review results in more detail.

Reviewer(s): [redacted]

Review Date: [redacted]

Case Report Prepared by: TROG Central Operations Office

Case Report Date: [redacted]
